# Evaluation and management of leukolysis-mediated pseudohyperkalemia in paediatric leukemic samples

**DOI:** 10.11613/BM.2022.010904

**Published:** 2022-02-15

**Authors:** Lourens Jan Peter Nonkes, Valérie de Haas, Hans Kemperman, Albert Huisman, Ruben Eduardus Antonius Musson, Wouter Marcel Tiel Groenestege

**Affiliations:** 1 Central Diagnostic Laboratory, Universitair Medisch Centrum Utrecht, Utrecht, The Netherlands; 2 Laboratory for Childhood Cancer Pathology, Prinses Máxima Center, Utrecht, The Netherlands

**Keywords:** leukolysis, pseudohyperkalemia, paediatric, leukocyte threshold

## Abstract

**Introduction:**

Leukolysis-related pseudohyperkalemia due to preanalytical procedures may lead to erroneous (or absence of) treatment based on an invalid lab test result. We aimed to obtain a leukocyte threshold above which leukolysis-related pseudohyperkalemia becomes clinical relevant. Secondly, temporal dynamics of treatment-induced leukocyte decrease were studied to allow tailored implementation of laboratory information system (LIS) decision rules based on the leukocyte threshold to avoid leukolysis-related pseudohyperkalemia.

**Materials and methods:**

Potassium results of AU5811 routine chemistry (Beckman Coulter, Brea, California, USA) and iStat point of care (POC) (Abbott Diagnostics, Chicago, Illinois, USA) analysers were compared, the latter method being insensitive to leukolysis caused by pre-analytical procedures. Potassium results were combined with leukocyte counts obtained using a Cell-Dyn Sapphire haematology analyser (Abbott Diagnostics, Santa Clara, California, USA), resulting in 132 unique data triplets. Regression analysis was performed to establish a leukocyte threshold. The Reference Change Value (√2 x Z x √(CV_a_^2^ + CV_i_^2^)) was used to calculate maximum allowable difference between routine analyser and POC potassium results (delta_max_ + 0.58 mmol/L). Temporal analysis on the treatment-induced leukocyte decrease was performed by plotting leukocyte counts in time for all patients above the threshold leukocyte count (N = 41).

**Results:**

Established leukocyte threshold was 63 x10^9^/L. Temporal analysis showed leukocyte counts below the threshold within 8 days of treatment for all patients.

**Conclusions:**

Based on performed analyses we were able to implement LIS decision rules to reduce pseudohyperkalemia due to preanalytical procedures. This implementation can contribute to a reduction in erroneous (or absence of) treatments in the clinic.

## Introduction

As a clinical laboratory that performs chemistry laboratory tests for the Princess Máxima Center for Pediatric Oncology (PMC) we regularly observe erroneous test results in paediatric leukemic samples due to leukolysis caused by pre-analytical procedures. These procedures, including pneumatic tube transport and sample centrifugation, can lead to lysis of fragile leukemic leukocytes in blood samples. This results in the release of intracellular constituents into the sample’s plasma fraction and can lead to incorrect test results. A prominent and clinically relevant deviation is leukolysis-related pseudohyperkalemia as described previously by us and others and can lead to erroneous (or absence of) treatment based on an invalid test result (*1*-*4*). Thus, higher leukocyte numbers are associated with larger deviances in potassium concentration. Although previously described for adult patient samples, to our knowledge these effects have never been systematically quantified in paediatric samples relative to a methodological approach that is insensitive to above described pre-analytical effects, like point of care (POC) testing (*4*). This whole blood analytical method can be performed at the bedside of the patient and circumvents the need for centrifugation and/or pneumatic tube transport.

The study aim was twofold: first, to determine the leukocyte threshold above which leukolysis-related pseudohyperkalemia becomes clinically relevant, and second, to establish the temporal dynamics of the treatment-induced leukocyte decrease to implement tailored laboratory information system (LIS) decision rules.

## Materials and methods

### Subjects

Test results over a period of 21 months of the PMC were extracted from the LIS (GLIMS v9, MIPS, Gent Belgium). This resulted in 47.513 laboratory orders (laborders) from 5335 unique paediatric patients (< 18 years). The study protocol was approved by the authors’ Institutional Review Board (METC 20-319C) and in accordance with the Helsinki Declaration.

### Methods

Potassium results of AU5811 routine chemistry (Beckman Coulter, Brea, USA) and iStat POC (Abbott Diagnostics, Santa Clara, USA) analysers were compared, the latter method being insensitive to leukolysis caused by pre-analytical procedures. Routine chemistry analyser samples were collected in lithium heparin blood collection tubes with gel separator (LH PST II, REF 367374; Becton, Dickinson and Company, Franklin Lakes, USA), send to the laboratory by pneumatic tube transport and centrifuged (5 minutes, 2000xg). For POC the first drop of blood after puncturing of the skin was collected in dry-sprayed balanced heparin capillaries MultiCap (Siemens, Erlangen, Germany) and immediately used for analysis.

Data were anonymized before further data processing and analysis using Jupyter Notebook v6.3.0 running on Python v3.6.7 (additional libraries: numpy v1.19.2, pandas v1.1.5, matplotlib 3.3.4, scipy 1.5.2, seaborn v0.11.1). Routine chemistry and POC analyser potassium results were compared in a within-patient manner and relative to the same leukocyte count. Thus, haematology analyser leukocyte counts Cell-Dyn Sapphire (Abbott Diagnostics, Santa Clara, USA) were linked to routine chemistry analyser potassium results if part of the same laborder. Leukocyte/routine chemistry potassium data pair was complemented with POC potassium result in case it was produced within two hours before/after the timestamp of the laborder. This resulted in 132 unique data triplets.

Linear regression analysis was performed in order to establish a leukocyte threshold value above which potassium results of the routine chemistry analyser deviated unacceptably from POC. Hereby the Reference Change Value (RCV) was used to calculate the maximum allowable difference between the routine analyser and POC potassium results (delta_max_). The RCV represents the smallest difference between sequential laboratory results associated with a true change in the patient and is defined as √2 x Z x √(CV_a_^2^ + CV_i_^2^), where Z is a number of standard deviations appropriate to the probability, CV*_a_* is the analytical coefficient of variation and CV*_i_* is coefficient of variation of within-subject biological variation (*5*). For Z-score 1.96 was chosen for *P <* 0.05, CV_a_ (0.86) was calculated based upon 4 measurements performed on 5 days using Biorad Liquid Assayed Mulitqual Premium quality control (Bio-Rad Laboratories Inc., Hercules, USA) at concentration 5.8 mmol/L. CV_i_ value (4.1) was obtained from (*6*). RCV (11.6%) was used in combination with the upper reference limit of potassium (5 mmol/L) to calculate delta_max_ (+0.58 mmol/L), as larger deviations at this concentration may trigger clinical action based on an erroneously elevated potassium result (*i.e.*, pseudohyperkalemia).

**Figure 1:**
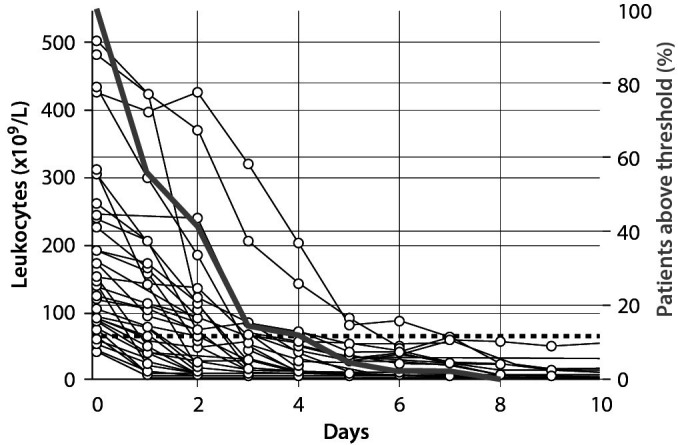
Temporal analysis of treatment-induced leukocyte decrease used to obtain a suitable time-window. Shown are patients that had a > 63 x10^9^/L leukocyte count at some time during treatment (N = 41). Horizontal black dotted line represents the leukocyte threshold (63 x10^9^/L). The bolded grey line reflects the percentage of patients above threshold at given day.

Temporal analysis of treatment-induced leukocyte decrease was performed by plotting leukocyte counts in time for all patients in our dataset that had a > 63 x10^9^/L leukocyte count (N = 41). For clarity purposes, in the case of multiple leukocyte measurements in a patient *per* day, results were averaged and represented as one data point for that day in Figure 1.

### Statistical analysis

Linear least-squares regression was used to calculate the leukocyte. Described confidence intervals were based on Student’s t-distribution. P < 0.05 was chosen as a level of statistical significance.

## Results

**Figure 2:**
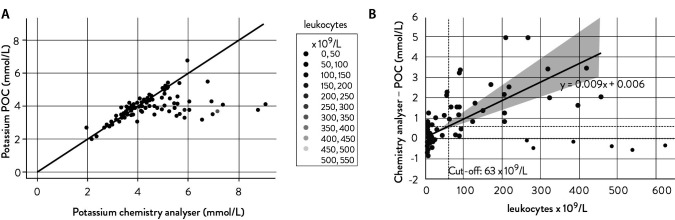
**(A)** Comparison between routine chemistry analyser and POC results for potassium. Black diagonal line represents x = y. Leukocyte count is denoted by hue color. (**B)** Linear regression analysis for potassium. Horizontal line – delta_max_, vertical line – leukocyte threshold (63 x10^9^/L). Black datapoints were excluded from the regression analysis. Hue represents 95% confidence interval (95% CI) Statistics: r = 0.76, *P* < 0.001, slope (+/- 95% CI): 0.01 (0.01 to 0.01), intercept (+/- 95% CI): 0.01 (-0.14 to 0.15). POC - point of care.

An evident leukocyte number-dependent increase in potassium concentration was observed for results produced by the routine chemistry analyser, compared to POC results (see Figure 2A). Next, we performed a linear regression analysis on the results in order to establish a leukocyte threshold value above which potassium results of the routine chemistry analyser deviated unacceptably from POC (see Figure 2B). Regression analysis showed that delta_max_ (*i.e.*, the maximum allowable difference between the routine analyser and POC potassium results; +0.58 mmol/L) was surpassed at a leukocyte count of > 63 x10^9^/L. In this analysis 6 outlier data points were excluded (see Discussion section).

Temporal analysis of the treatment-induced leukocyte decrease indicated that all patients showed a leukocyte reduction below the threshold of 63 x10^9^/L within 8 days (see Figure 1).

## Discussion

Here we established a leukocyte threshold of 63 x10^9^/L above which pseudohyperkalemia may become a clinical concern. Secondly, we showed that treatment-induced leukocyte reductions below the threshold can generally be observed within the first 8 days. Based on these findings diagnostic procedures were modified by implementing a number of automated decision rules, in order to avoid pseudohyperkalemia due to high leukocyte numbers. As the frequency at which this phenomenon occurs in the paediatric PMC population is considerable, we decided to use POC testing for all potassium requests at the PMC. However, if also other chemistry analytes, not covered by the POC analyser, are ordered, potassium is measured on the routine chemistry analyser in order to reduce the number of blood draws and the total required blood volume. However, if a > 63 x10^9^/L leukocyte count is reported, potassium is redirected back to POC testing irrespective of whether additional chemistry analytes are ordered. This rule is active until *1)* a leukocyte count below 63 x10^9^/L is reported, or *2)* an 8-day time window has passed. This time window is based on the observed treatment-induced leukocyte reduction rate in leukemic paediatric patients (see Figure 1).

As indicated in the results section, 6 outliers were excluded from regression analysis. Outliers had > 63 x10^9^/L leukocyte counts but showed smaller deviations than delta_max_. This may be explained by the observation that not all leukemic variants are associated with fragile leukocytes and thereby leukolysis-related pseudohyperkalemia (*4*). As the dataset was anonymized, we could not investigate this possibility. Another explanation may be that POC samples were haemolytic, leading to elevated potassium concentration in these samples and thereby smaller differences relative to routine chemistry analyser results. As the POC apparatus does not allow the quantification of haemolysis we can neither confirm nor exclude this possibility. We decided to exclude these 6 deviating datapoints from the regression analysis as both above explanations are not relevant for establishing a leukocyte threshold to counter leukolysis-related pseudohyperkalemia. For 8 other datapoints delta_max_ was already surpassed at < 63 x10^9^/L leukocytes. This may be (partly) explained by haemolysis in the routine analyser sample. Routine chemistry analyser quantifies haemolysis in measured samples. We confirmed haemolysis in 7 of these 8 routine analyser samples (data not shown).

Due to dataset anonymization, a study limitation is that we could not discriminate between different types of leukaemia in the analysis, whereas studies have shown that pseudohyperkalemia is predominantly associated with specific leukaemia variants (*4*). Because of the paediatric nature of included patients it is likely that most leukaemia variants were of acute lymphocytic origin, the most common paediatric leukaemia variant (*7*). As such, this study’s leukocyte threshold may not translate well to other patient populations in which acute lymphocytic leukaemia occurrence is less frequent, like in adults. Interestingly, Ranjitkar *et al.* who included adults and multiple leukaemia variants, reported a 50 x10^9^/L leukocyte threshold above which patients were at risk for pseudohyperkalemia, which is not that different from the present study’s threshold, albeit somewhat lower (*4*). This lower threshold may be the result of the inclusion of a relatively large number of patients with chronic lymphocytic leukaemia, a leukaemia variant associated with leukocyte fragility (*2*, *4*, *8*).

Another factor that likely affects leukolysis rate is the patient’s specific treatment phase. Also here, because of anonymization we were not able to include this information and therefore is a limitation of this study.

In practice knowledge of the leukemic variant or treatment might help to fine-tune and further personalize the management of leukolysis-related laboratory artifacts like pseudohyperkalemia. However, this prompts the initiation of additional studies to determine specific leukocyte cut-off values for different leukemic variants and treatments.

In conclusion, based on performed analyses we were able to implement tailored LIS decision rules to reduce pseudohyperkalemia due to pre-analytical procedures. This can contribute to a reduction in erroneous (or absence of) treatments in the clinic.

## Data Availability

The data generated and analysed in the presented study are available from the corresponding author on request.
